# Supplementary material for: Extent of N-Terminus Folding of Semenogelin 1 Cleavage Product Determines Tendency to Amyloid Formation
Source: Int J Mol Sci. 2023 May 18;24(10):8949. doi: 10.3390/ijms24108949 (PMC10219109; doi:10.3390/ijms24108949)
Supplement: Supplementary file 1 [file ijms-24-08949-s001.zip › ijms-2332049-supplementary.pdf]

Extent of N-Terminus Folding of Semenogelin 1 Cleavage Product Determines Tendency to Amyloid Formation

Daria A. Osetrina [1], Aleksandra M. Kusova [1,2], Aydar G. Bikmullin [1,3], Evelina A. Klochkova [1,3], Aydar R. Yulmetov [1], Evgenia A. Semenova [1], Timur A. Mukhametzyanov [1], Konstantin S. Usachev [3,4], Vladimir V. Klochkov [1,*] and Dmitriy S. Blokhin [1]

[1] NMR Laboratory, Medical Physics Department, Institute of Physics, Kazan Federal University, Kremlevskaya Str., 18, Kazan 420008, Russia; d.sanchugova@yandex.ru (D.A.O.); alexakusova@mail.ru (A.M.K.); aydar.bikmullin@gmail.com (A.G.B.); evelina.klochkova@gmail.com (E.A.K.); ajulmeto@gmail.com (A.R.Y.); jzh.sem@mail.ru (E.A.S.); timur.mukhametzyanov@kpfu.ru (T.A.M.); dmitr.blokhin@gmail.com (D.S.B.)
[2] Kazan Institute of Biochemistry and Biophysics, FRC Kazan Scientific Center, Russian Academy of Sciences, Kazan 420111, Russia
[3] Laboratory of Structural Biology, Institute of Fundamental Medicine and Biology, Kazan Federal University, Kazan 420021, Russia; konstantin.usachev@kpfu.ru
[4] Laboratory for Structural Analysis of Biomacromolecules, Federal Research Center "Kazan Scientific Center of Russian Academy of Sciences", Kazan 420111, Russia
* Correspondence: vladimir.klochkov@kpfu.ru; Tel.: +7-(843)-233-7634

Supplementary Materials

Part.1. Experimental of 2D NMR data:

2D ^{1}H-^{1}H TOCSY

(TOCSY mixing time d9 = 100ms; number of scans ns= 40; spectral width sw=12ppm)

2D ^{1}H-^{1}H NOESY

(mixing time t_m = 300ms; ns=48; sw=12ppm

2D ^{1}H-^{15}N HSQC (Heteronuclear Single Quantum Coherence)

(ns=128; sw=^{1}H 12ppm/^{15}N 35ppm)

2D ^{1}H-^{13}C HSQC

(ns=64; sw= ^{1}H 12ppm/^{13}C 165ppm)

2D ^{1}H-^{13}C HMBC (Heteronuclear Multiple Bond Correlation)

(ns=128; sw= ^{1}H 12ppm/^{13}C 200ppm)

2D ^{1}H-^{13}C HSQC-TOCSY

(ns=64; sw= ^{1}H 12ppm/^{13}C 165ppm)

Part 2. Structural NMR studies

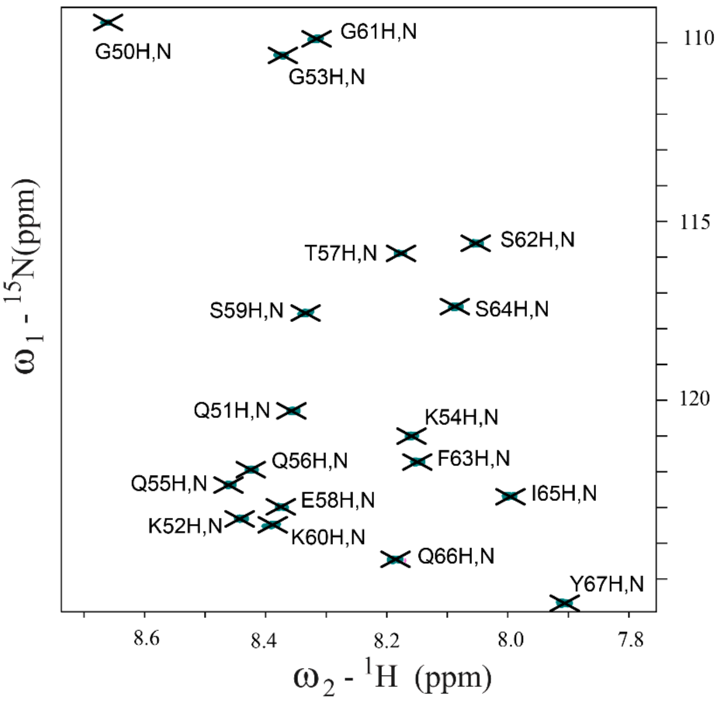

Figure S1. Fragment of 2D ^{1}H-^{15}N HSQC NMR spectrum of SEM1(49–67) in aqua solution at 298K.

Table S1. Distance constraints of SEM1(45–67) peptide from analysis of ^{1}H-^{1}H NOESY NMR spectra.

	Pair of atoms	Range of internuclear distances, Å		Pair of atoms	Range of internuclear distances, Å		Pair of atoms	Range of internuclear distances, Å
1	Q46 HN – Q46 HA		49	Q66 HN – Q66 HG#		97	K54 HN – 50G HA#	[1.80; 5.50]
2	Q46 HN – S49 HB#		50	Y67 HN – Y67 HB#		98	Y48 HN – H47 HA	
3	Q46 HN – S49 HN		51	Y67 HA – Y67 HB#	[1.80; 5.00]	99	K60 HN – S59 HA	[1.80; 3.35]
4	Q46 HB# – Q66 HG#		52	Y67 HD# – Y67 HA		100	Y67 HN – Q66 HA	
5	H47 HB# – H47 HA		53	Y67 HD# – Y48 HA		101	Y48 HB# – Y48 HD#	
6	H47 HN – H47 HA		54	Q46 HN – Q46 HB#		102	Y48 HN – Y48 HB#	
7	H47 HN – H47 HB#		55	H47 HN – H47 HE1		103	S49 HA – S49 HN	
8	H47 HN – Y48 HN		56	Y48 HA – Y48 HB#		104	S49 HN – S49 HB#	
9	H47 HD2 – Y67 HB#		57	K54 HE# – F63 HA		105	50G HN – 50G HA#	
10	H47 HN – Y67 HB#		58	E58 HN – E58 HG#		106	Q51 HN – Q51 HB#	
11	Y48 HN – Y48 HD#		59	G61 HA# – Q55 HN		107	Q51 HN – Q51 HA	
12	Y48 HD# – Y48 HA		60	F63 HB# – F63 HA		108	Q51 HN – 50G HN	
13	S49 HN – Y67 HN		61	S64 HN – F63 HB#	[1.80; 6.00]	109	K52 HN – K52 HB#	
14	S49 HA – E58 HB#		62	S64 HN – F63 HD#		110	K52 HN – Q51 HB#	
15	Q51 HG# – Q51 HN		63	S64 HN – I65 HN		111	K54 HN – K54 HB#	
16	K52 HB# – K52 HG#		64	I65 HN – I65 HG1#		112	K54 HN – K54 HD#	
17	K52 HE# – Y48 HA		65	I65 HG2# – I65 HD1#		113	K54 HN – K54 HA	
18	K52 HG# – I65 HG1#		66	Y67 HD# – Y67 HB#		114	Q55 HB# – Q55 HN	
19	G53 HA# – G53 HN		67	Y67 HD# – Y67 HN		115	Q55 HN – K54 HB#	
20	K54 HD# – K54 HA		68	Y67 HB# – H47 HA		116	Q55 HN – T57 HN	
21	K54 HN – K54 HG#		69	Y48 HN – Y48 HA		117	Q56 HN – Q56 HB#	
22	K54 HN – K52 HN		70	S49 HB# – S49 HA		118	Q56 HB# – Q56 HG#	
23	Q55 HN – K54 HD#		71	K52 HG# – K52 HD#		119	Q56 HG# – Q56 HE2#	
24	Q56 HA – Q56 HB#	[1.80; 5.00]	72	K52 HA – K52 HN		120	Q56 HE2# – Q46 HG#	
25	Q56 HA – Q56 HN		73	Q55 HA – Q55 HN		121	Q56 HN – T57 HN	
26	Q56 HN – K54 HN		74	S59 HN – S59 HA		122	Q56 HA – T57 HN	[1.80; 3.30]
27	Q56 HG# – Q56 HN		75	K60 HG# – K60 HD#		123	T57 HN – T57 HB	
28	E58 HN – K60 HB#		76	K60 HA – K60 HN	[1.80; 2.70]	124	T57 HA – T57 HB	
29	E58 HN – K60 HG#		77	G61 HN – G61 HA#		125	T57 HA – K60 HB#	
30	S59 HN – S59 HB#		78	S64 HB# – S64 HA		126	E58 HN – E58 HB#	
31	S59 HB# – K60 HN		79	S64 HB# – S64 HN		127	E58 HB# – E58 HG#	
32	K60 HZ# – K60 HE#		80	S64 HN – S64 HA		128	K60 HB# – K60 HG#	
33	K60 HN – K60 HB#		81	Q66 HB# – Q66 HG#		129	K60 HN – T57 HB	
34	K60 HA – K60 HB#		82	H47 HN – Q46 HA		130	G61 HN – E58 HA	
35	S62 HB# – S62 HA		83	G53 HN – K54 HN		131	S62 HA – S62 HN	
36	S62 HN – F63 HB#		84	G61 HA# – S62 HN		132	S62 HN – S62 HB#	
37	S62 HN – K54 HE#		85	I65 HA – I65 HG2#		133	S62 HA – F63 HN	
38	F63 HD# – F63 HB#		86	I65 HG2# – I65 HN	[1.80; 6.50]	134	F63 HN – F63 HA	
39	F63 HN – F63 HD#		87	I65 HA – I65 HD1#		135	F63 HN – S64 HN	
40	F63 HN – F63 HB#		88	I65 HG1# – I65 HG2#		136	F63 HN – K54 HE#	
41	F63 HN – S62 HB#		89	T57 HN – T57 HG2#		137	S64 HN – F63 HA	
42	F63 HN – S62 HN		90	T57 HA – T57 HG2#	[1.80; 5.50]	138	I65 HA – I65 HB	
43	F63 HD# – K54 HE#		91	I65 HB – I65 HD1#		139	I65 HB – I65 HG1#	
44	S64 HN – S62 HB#		92	K60 HN – T57 HG2#		140	I65 HA – I65 HN	
45	I65 HA – I65 HG1#		93	T57 HG2# – T57 HB		141	I65 HA – Q66 HN	
46	I65 HN – I65 HB		94	I65 HB – I65 HG2#	[1.80; 3.20]	142	Y67 HD# – K52 HE#	
47	Q66 HA – Q66 HB#		95	I65 HD1# – I65 HG1#		143	Y67 HD# – Y48 HB#	
48	Q66 HN – Q66 HB#		96	Q56 HN – K52 HB#	[1.80; 5.99]			

Table S2. Distance constraints of SEM1(49–67) peptide from analysis of ^{1}H-^{1}H NOESY NMR spectra.

	Pair of atoms	Range of internuclear distances, Å		Pair of atoms	Range of internuclear distances, Å		Pair of atoms	Range of internuclear distances, Å
1	K52 H – Q66 H		25	Q51 HG# – Q51 HB3		49	I65 HG1# – I65 HD1#	[1.80; 3.80]
2	K52 HB# – K52 HG#		26	K52 HD# – K52 HB#		50	I65 HD1# – K52 HG#	
3	G53 HA# – K54 H		27	K52 HD# – K52 HE#		51	K52 HZ# – Q55 HE2#	
4	K54 HB# – K54 HG#		28	K52 HE# – K52 HG#		52	K54 HD# – K54 HG#	
5	K54 HB3 – K54 HD#		29	K52 HZ# – K52 HE#		53	Q56 H – Q56 HA	[1.80; 2.70]
6	K54 HZ# – Q55 HG#		30	K54 HB3 – K54 HD#		54	S59 H – S59 HA	
7	Q55 HG# – Q55 HB#		31	Q55 HG# – Q55 HB#		55	Y67 HE# – Y67 HD#	
8	Q56 H – Q56 HB#		32	Q56 HA – Q56 HG#		56	S49 HB# – S49 HA	
9	E58 HG# – E58 HA		33	Q56 HB# – Q56 HG#		57	50G HA# – 50G H	
10	K60 HB# – K60 HG#		34	T57 HB – T57 HA	[1.80; 3.30]	58	Q51 HB3 – Q51 HA	
11	G61 H – G61 HA#		35	E58 HG# – E58 HB#		59	E58 H – T57 HB	
12	S62 HB# – S62 H		36	S59 HA – S59 HB#		60	S59 HB# – S59 H	
13	F63 H – F63 HA	[1.80; 5.00]	37	K60 HB3 – K60 HG#		61	S62 H – S62 HA	[1.80; 6.00]
14	F63 HB3 – F63 HA		38	K60 HE# – K60 HD#		62	F63 HD# – F63 HB3	
15	F63 HD# – F63 HB#		39	K60 HZ# – F63 HE#		63	S64 H – S64 HA	
16	I65 H – I65 HB		40	I65 H – S64 HA		64	S64 HB# – I65 HA	
17	I65 HG1# – K52 HG#		41	I65 HA – Q66 H		65	I65 HD1# – I65 HG2#	
18	Q66 H – Q66 HA		42	Q66 H – Q66 HA		66	Q66 HG# – Q66 HA	
19	Q66 HG# – Q66 HB#		43	Q66 HG# – Q66 HB#		67	Y67 HD# – Y67 HB#	
20	Y67 H – Q66 HA		44	H56 A– H57 G2#				
21	Y67 HA – Y67 H		45	H57 G2# – H57 B				
22	Y67 HD# – Y67 HE#		46	I65 HA – I65 HG2#	[1.80; 5.50]			
23	Y67 HD# – Y67 HB3		47	I65 HG2# – I65 HB				
24	Y67 HD# – Y67 HA		48	I65 HD1# – I65 HB				

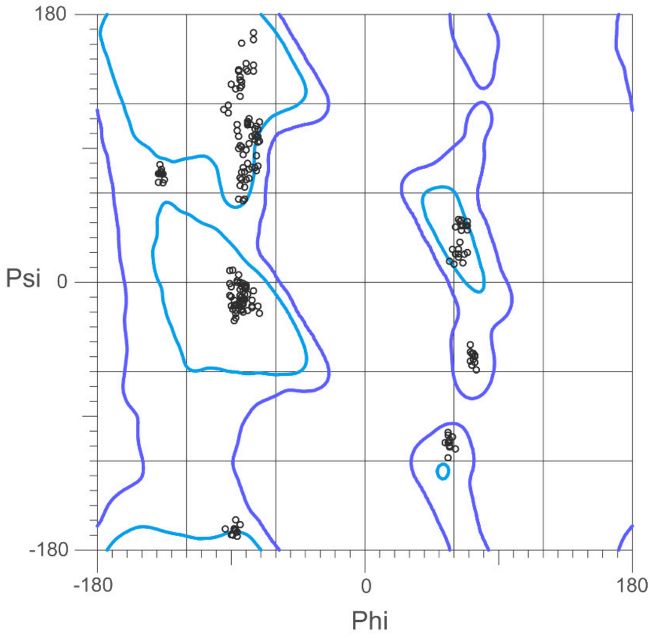

Figure S2. Ramachandran plot of SEM1(45–67) conformers. All signals of SEM1(45–67) in Ramachandran plot were in favored regions (80.1 %) and allowed regions (19.9%).

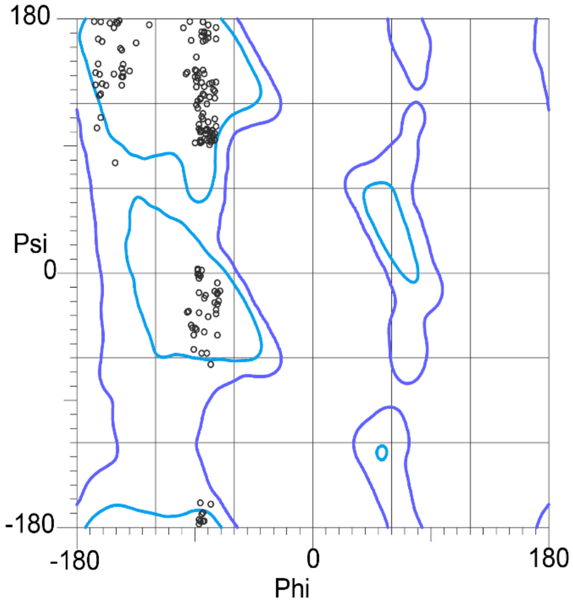

Figure S3. Ramachandran plot of SEM1(49–67) conformers. All signals of SEM1(49–67) in Ramachandran plot were in favored regions (94.1 %) and allowed regions (5.9%).

Table S3. DSSP analysis of SEM1(45–67) structure. Secondary structure: c is random coil; S is bend; T is turn; G is 3_{10}-helix.

Residue number	45	46	47	48	49	50	51	52	53	54	55	56	57	58	59	60	61	62	63	64	65	66	67
Amino acid sequence	G	Q	H	Y	S	G	Q	K	G	K	Q	Q	T	E	S	K	G	S	F	S	I	Q	Y
Secondary structure	c	c	S	S	T	T	T	S	S	S	S	S	c	G	G	G	G	S	S	c	c	c	c

Table S4. DSSP analysis of SEM1(49–67) structure. Secondary structure: c is random coil; S is bend.

Residue number	49	50	51	52	53	54	55	56	57	58	59	60	61	62	63	64	65	66	67
Amino acid sequence	S	G	Q	K	G	K	Q	Q	T	E	S	K	G	S	F	S	I	Q	Y
Secondary structure	c	c	S	c	S	c	c	c	S	S	S	c	c	c	S	c	c	c	c

Part 3. Analysis of SEM1(45–67) and SEM1(49–67) surfaces

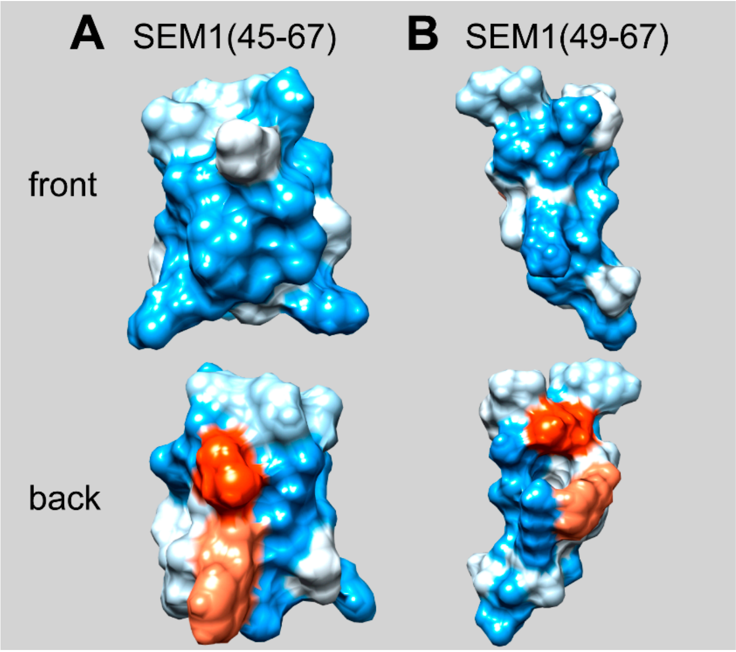

Figure S4. The calculated hydrophobic surfaces of SEM1(45–67) (a) and SEM1(49–67) (b) by UCSF Chimera program. Red color is hydrophobic regions, blue color represents hydrophilic regions.

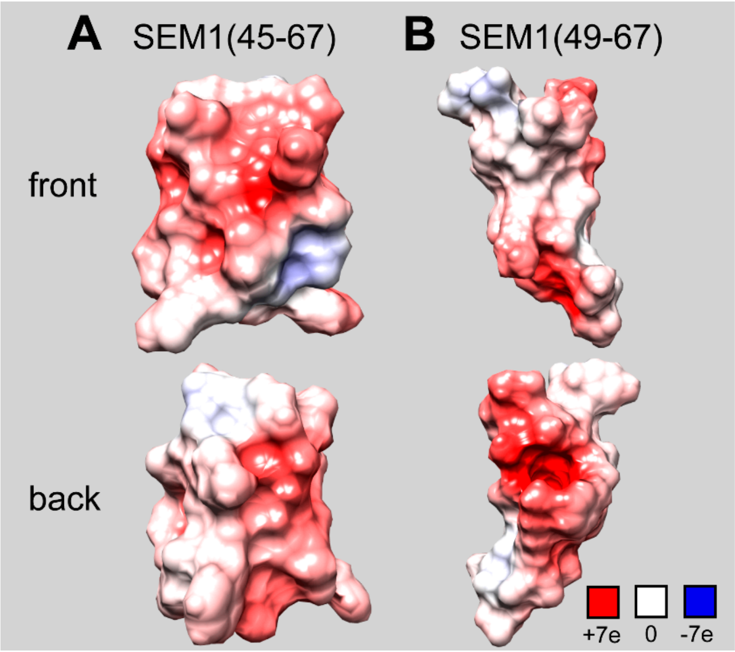

Figure S5. The calculated electrostatic surfaces of SEM1(45–67) (a) and SEM1(49–67) (b) by APBS-PDB2PQR software suite (https://server.poissonboltzmann.org). Red color represents positive charged regions, blue color is negative charges ones.

Part 4. ThT fluorescence

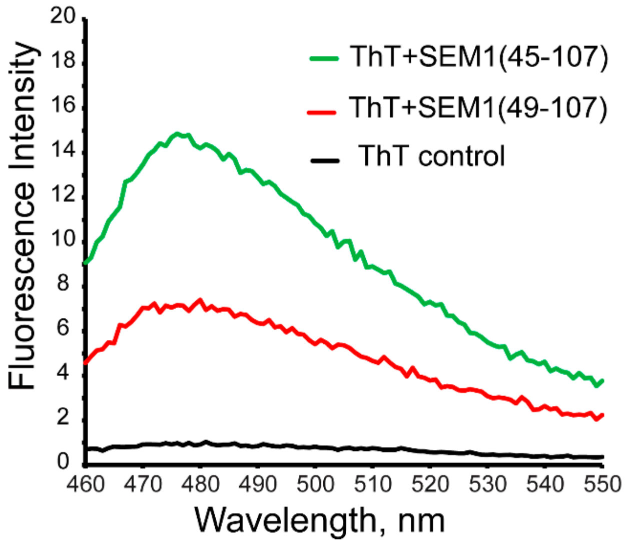

Figure S6. Fluorescence spectra of Thioflavin T (ThT) (black line), SEM1(49–107) with ThT (red line), and SEM1(45–107) with ThT (green line). Spectra were recorded 30 minutes after purification of peptides.

Part 5. MD simulation

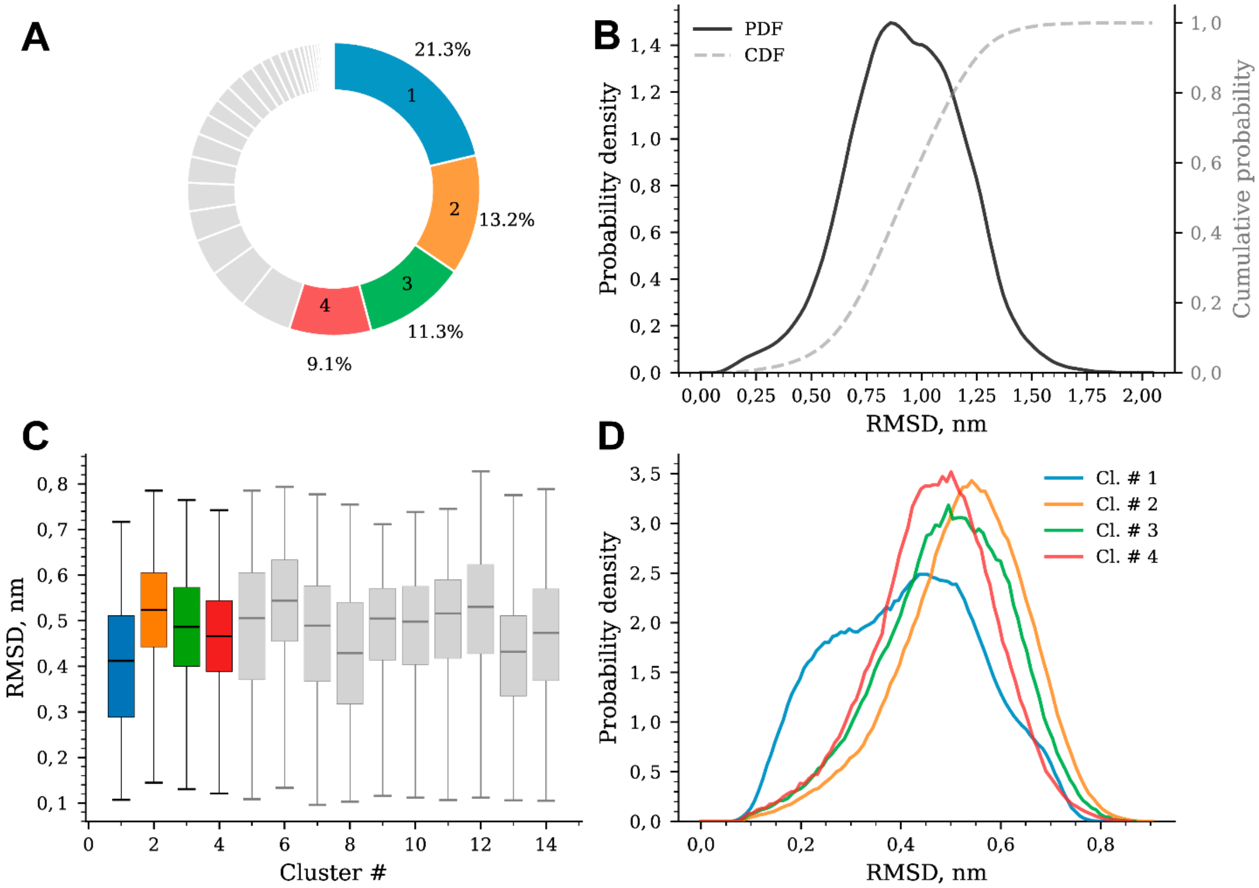

Figure S7. (A) Donut plots quantifying the conformational states of SEM1(45–107) peptide. The diagram shows the fractional occupancies of the four most populated clusters. Each slice represents a distinct state. RMS deviation of Cα-Cα atom-pair distances were used to define the distance between structures for GROMOS clustering. (B) Probability distribution function (PDF) and cumulative distribution function (CDF) for RMS deviation of Cα-Cα atom-pair distances. Frames with 10 ps time step of full MD trajectory were used for calculations. (C) Box plot of RMSD distributions of Cα-Cα atom-pair distances for frames assigned to individual clusters. Every box is shown from the first quartile to the third quartile of RMSD distribution. A horizontal line goes through the box at the median. The whiskers go from each quartile to the minimum or maximum. (D) Probability distribution function (PDF) for RMS deviation of Cα-Cα atom-pair distances of the four most populated clusters.

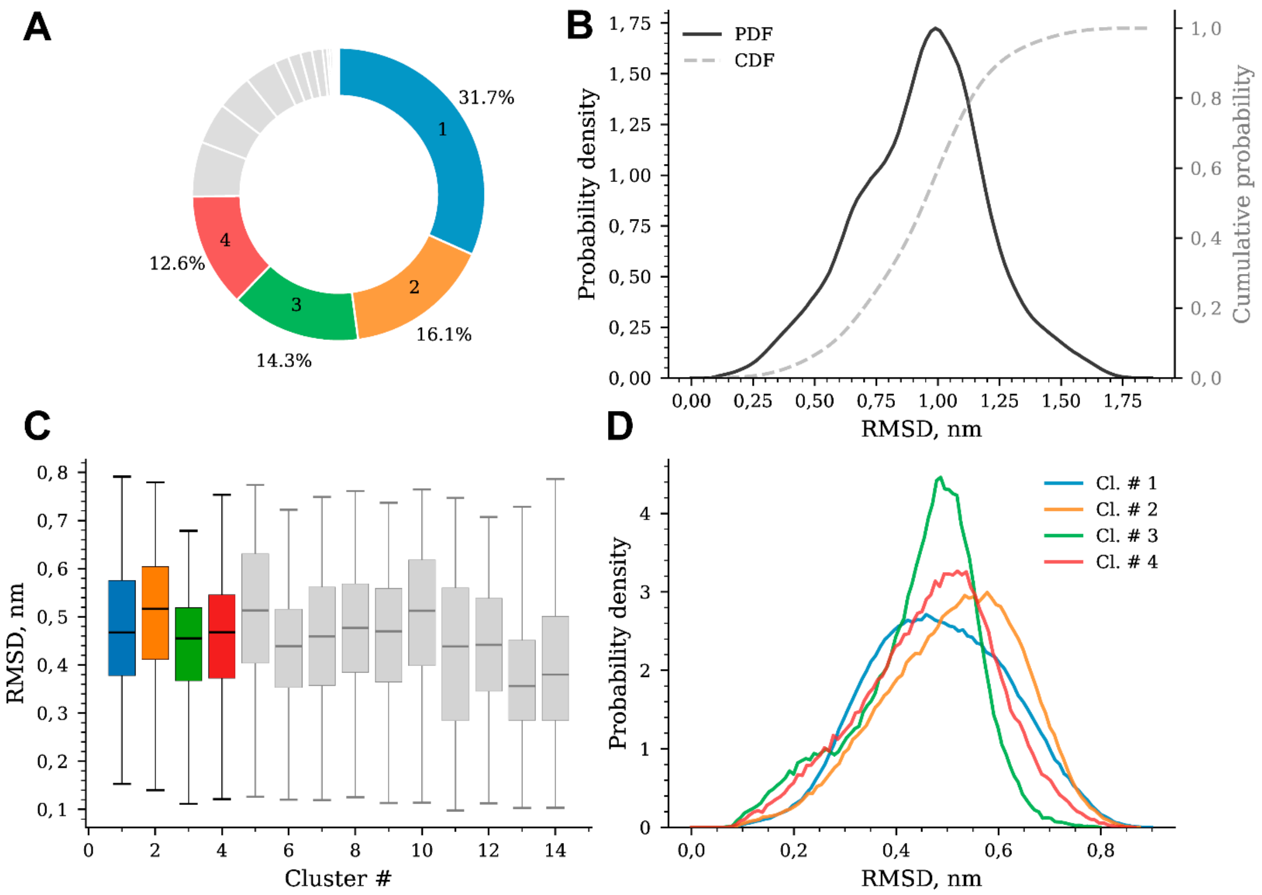

Figure S8. (A) Donut plots quantifying the conformational states of SEM1(49–107) peptide. The diagram shows the fractional occupancies of the four most populated clusters. Each slice represents a distinct state. RMS deviation of Cα-Cα atom-pair distances were used to define the distance between structures for GROMOS clustering. (B) Probability distribution function (PDF) and cumulative distribution function (CDF) for RMS deviation of Cα-Cα atom-pair distances. Frames with 10 ps time step of full MD trajectory were used for calculations. (C) Box plot of RMSD distributions of Cα-Cα atom-pair distances for frames assigned to individual clusters. Every box is shown from the first quartile to the third quartile of RMSD distribution. A horizontal line goes through the box at the median. The whiskers go from each quartile to the minimum or maximum. (D) Probability distribution function (PDF) for RMS deviation of Cα-Cα atom-pair distances of the four most populated clusters.
